# Researching interactions between humans and machines: methodological challenges

**DOI:** 10.1007/s11616-022-00759-3

**Published:** 2022-11-17

**Authors:** Esther Greussing, Franziska Gaiser, Stefanie Helene Klein, Carolin Straßmann, Carolin Ischen, Sabrina Eimler, Katharina Frehmann, Miriam Gieselmann, Charlotte Knorr, Angelica Lermann Henestrosa, Andy Räder, Sonja Utz

**Affiliations:** 1 Institute for Communication Science, TU Braunschweig, Bienroder Weg 97, 38106 Braunschweig, Germany; 2 Leibniz-Institut für Wissensmedien, Schleichstraße 6, 72076 Tübingen, Germany; 3 Computer Science Institute, University of Applied Sciences Ruhr West, Lützowstraße 5, 46236 Bottrop, Germany; 4 Amsterdam School of Communication Research, University of Amsterdam, Nieuwe Achtergracht 166, 1018 WV Amsterdam, The Netherlands; 5 Department of Communication Studies, Heinrich Heine University Düsseldorf, Ulenbergstraße 127, 40225 Düsseldorf, Germany; 6 Department for Communication and Media Studies, Leipzig University, Nikolaistraße 27–29, 04109 Leipzig, Germany; 7 Department of Media Studies, University of Rostock, August-Bebel-Str. 28, 18055 Rostock, Germany

**Keywords:** Human-machine communication, Human-machine interaction, Human-computer interaction, Methodological challenges, Communicative agents, Communication

## Abstract

Communication scholars are increasingly concerned with interactions between humans and communicative agents. These agents, however, are considerably different from digital or social media: They are designed and perceived as life-like communication partners (i.e., as “communicative subjects”), which in turn poses distinct challenges for their empirical study. Hence, in this paper, we document, discuss, and evaluate potentials and pitfalls that typically arise for communication scholars when investigating simulated or non-simulated interactions between humans and chatbots, voice assistants, or social robots. In this paper, we focus on experiments (including pre-recorded stimuli, vignettes and the “Wizard of Oz”-technique) and field studies. Overall, this paper aims to provide guidance and support for communication scholars who want to empirically study human-machine communication. To this end, we not only compile potential challenges, but also recommend specific strategies and approaches. In addition, our reflections on current methodological challenges serve as a starting point for discussions in communication science on how meaning-making between humans and machines can be investigated in the best way possible, as illustrated in the concluding section.

## Introduction

Communication today takes place not only between humans but also between humans and machines (cf. Guzman 2018). Advances in artificial intelligence (AI) and automation have produced various technologies that transcend their function as mere channels or mediums: By exchanging messages with their users, technologies such as chatbots, voice assistants or social robots become active participants in the communication process. This results in communication contexts in which people no longer speak *through* technology but *to* and *with* it, interpreting it as life-like social actors (cf. Fortunati and Edwards 2021; Guzman and Lewis 2020). The conceptualization of technology as a “communicative subject” (Guzman 2018, p. 17) significantly departs from traditional understanding of technology in computer-mediated communication (CMC), leading to a new field of human-machine communication (HMC) that centers on the creation of meaning among humans and machines (cf. Guzman 2018; Spence 2019).

To adequately study this meaning-making, scholars have repeatedly argued for a critical revision of the existing theoretical and methodical repertoire, as this was originally developed for communication between humans (cf. Guzman 2018). While the theoretical aspects have received much scholarly attention (cf. Gambino and Liu 2022; Gibbs et al. 2021; Gambino et al. 2020; Westerman et al. 2020), the newly emerging communicative relations between humans and machines also come with methodological implications that need to be reflected upon. Most fundamentally, the objects of study are communicative agents that can independently execute self-directed behaviors, shifting control from the human to the machine (cf. Banks 2019, p. 364; Hepp 2020). Compared to digital or social media as communication channels or platforms, communicative agents provide more autonomous, personalized, and, ultimately, “human” forms of communication that considerably shape the interactions with users. Drawing on the peculiarities of these new technologies, this paper aims to document, discuss, and evaluate challenges that typically arise for communication scholars when planning and designing an empirical study on interactions between humans and communicative agents—particularly chatbots, voice assistants, and social robots.

Understanding how people interact with communicative agents is a primary objective of HMC research (cf. Guzman and Lewis 2020). It involves questions such as how people perceive technology in light of social affordances (cf. Reeves and Nass 1996; Sundar 2008), how they conceptualize its hybrid status (cf. Etzrodt and Engesser 2021; Weidmüller 2022) or establish a longer-term relationship with them (cf. Mavrina et al. 2022). Empirical investigations of these issues mostly rely on established approaches from human-human communication while considering that the unique characteristics of communicative agents require methodological adjustments (cf. Richards et al. 2022). In what follows, we focus on these adjustments and provide an overall overview of challenges that arise in experiments and field studies, which are two methodological approaches that play a key role in current HMC scholarship (cf. Liu et al. 2022; Richards et al. 2022).

Communicative agents can take many forms. In this paper, we consider chatbots, voice assistants, and social robots. As such, we are concerned with research objects that are designed as stand-alone communicators that can communicate via different modalities (i.e., text, voice, gestures, or movement) and take on different degrees of embodiment. Embodiment here means that the communicative agent can be recognized by a material artifact that operates in the physical world, ranging from minimalist smart speaker casings to the bodies of human-like (humanoid) robots (cf. Gunkel 2020, p. 206 f.). Although chatbots, voice assistants, and social robots are all based on natural language interactions, from a methodological perspective, distinguishing their main communication modality is essential. In both experiments and field studies, the study of speech-based interactions has different requirements on the technical setup and thus on the skills and resources of the researchers than those of text-based interactions. Written and spoken language also contain distinct social cues that need to be considered, for example, when controlling for intervening variables in experimental designs. To make these methodological implications visible, we explicitly limit our reflections to chatbots, voice assistants, and social robots.

By inventorying and critically reflecting on the methodical repertoire, this paper contributes to HMC research from a communication perspective in at least two ways. First, it is intended to guide and support communication scholars, especially novices in the field of HMC, by making them aware of the unique characteristics of communicative agents as research objects, which might make it difficult to directly adopt classical research designs (cf. Richards et al. 2022). We thereby aim to contribute to transparency in the field by not only comprehensively collecting and sharing potential challenges but also recommending strategies. The latter, however, always needs to be seen in light of the respective research question and context, as there is no one-fits-all solution. Second, this paper acknowledges that technology is subject to constant change (cf. Guzman and Lewis 2020), which implies that its empirical investigation also needs to be adaptable. Hence, we aim to gather the scattered empirical literature and introduce approaches from different disciplinary fields to communication science. In this sense, our reflections not only bring together what has previously been discussed separately for specific methods (cf. Riek 2012; Schmidt et al. 2021; Porcheron et al. 2020), types of communicative agents (cf. Woods et al. 2006; Ren et al. 2019; Walters et al. 2005), or disciplinary fields (cf. Baxter et al. 2016; Eyssel 2017), but can also serve to initiate methodological advancements in communication science that can lead to a more complete investigation of interactions between humans and communicative agents. Finally, the lessons learned about the empirical practices of researching interactions between humans and machines are displayed in a summary table (see Table 1), aiming to help communication scholars access this new field of research even more readily.

**Table 1:** Benefits and downsides of experiments and field studies in HMC research

			Benefits	Limitations
Experiments	With simulated interactions	Pre-recorded material	Manipulation of specific agent characteristics Large samples Affordable Easier to implement for less embodied agents	Participants are passive observers (no interaction)
		Wizard of Oz	Interaction between participant and agent Full control over the interaction Experiments on future agent skills Promote replication	Not free of human bias Ethical problems (deception of participants) Time- and resource-consuming Difficult to control multiple modalities at once
	With non-simulated interactions	Self-created agents	Authentic interaction Control over the interaction	High effort Programming skills required
		Commercially available agents	Authentic interaction Fast, easy, and cost-effective obtainability Less error-prone	Less control over the material and the interaction Depends on available commercially agents Previous experience with the agent as moderator
Field Studies			Real life data from authentic interactions in natural environments High external validity Interactions over time can be observed Large datasets through donation or collaboration	Less control Time- and resource-consuming High organizational efforts, e.g., collaborations Well-functioning technology and support needed Specific challenges for private vs. public settings

**Fig. 1:**
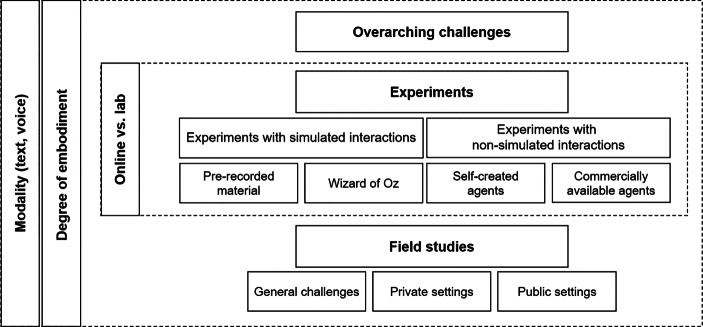
Structure of the paper

In the following, we first address overarching challenges that arise irrespective of the methodological approach. We then discuss challenges associated with experimental designs and field studies, starting with online and lab experiments (distinguishing between experiments with simulated and non-simulated interactions), followed by field studies in private and public settings (see Fig. 1 for an overview of the paper’s structure). This collection of methodological challenges is the result of an extensive literature review conducted by the first three authors and critically assessed and completed by the remaining co-authors, all academic professionals in different fields of HMC.

## Overarching challenges when studying interactions between humans and communicative agents

The characteristics of communicative agents as research objects imply several overarching challenges in the design and implementation of empirical studies. First, although the proliferation of communicative agents such as chatbots, voice assistants, and social robots has increased steadily over the past few years (cf. Wiederhold 2021), *interacting* with them has not yet become a daily practice everywhere (Gentsch 2020; RMS 2020). As a result, large samples of experienced users are difficult to obtain, even more so when recruitment is geographically constrained, as is the case with laboratory or field studies. A considerable amount of HMC research therefore relies on studies among people who have never turned to a communicative agent like a voice assistant (cf. Beirl et al. 2019) or a social robot (Edwards et al. 2019) before. While these studies provide valuable insights into first encounters between humans and machines under equal conditions, they may come with novelty effects that compromise the generalizability of the results (cf. Croes and Antheunis 2021; Weidmüller 2022). Small sample sizes, as seen in much HMC research (cf. Rapp et al. 2021, p. 6), also pose a threat to statistical power and the associated detection of actual effects in the sample.

Second, there are only a few validated scales specifically developed to measure the meaning-making between humans and machines (see e.g., Kim and Sundar (2012, p. 249) for mindless anthropomorphism). Hence, scales that have originally been developed for interaction processes between humans or between humans and computers are applied to research on chatbots, voice assistants, or social robots. These communicative agents, however, are perceived as something in-between humans and computers (“personified things”, Etzrodt and Engesser 2021, p. 73), which raises the question of whether scales initially developed for other contexts can be adequately transferred to evaluate a communicative agent (cf., for a comprehensive approach Eyssel 2017). One prominent example is the scale for social presence (cf. Gefen and Straub 2003), which was originally developed to assess social presence on an e‑commerce website (cf. Pitardi and Marriott 2021). Concepts such as trust have also been applied to and operationalized for both humans and technology in the past (cf. Mayer et al. 1995, p. 717; McKnight et al. 2011, p. 13). For communicative agents, however, both groups of items seem applicable, considering that people perceive different agents as social, anthropomorphic, and yet clearly non-human. Here, first attempts have been made to combine trust scales in humans and technology to create a multidimensional trust model in social robots and voice assistants that matches their hybrid perception (cf. Weidmüller 2022).

Finally, people’s low levels of experience with communicative agents together with the agents’ unique ontology require special attention to how the agent is presented to study participants. There is evidence that how a robot is described might influence users’ perception and evaluation of the robot, making it an important confounding aspect of the study design (Rosenthal-von der Putten et al. 2017; Mara and Appel 2015). Given that individuals have informal theories about technological systems even before they have ever engaged with them (cf. Gruber et al. 2021), using specific metaphors (cf. Khadpe et al. 2020) or terms such as artificial intelligence (cf. Alizadeh et al. 2021) when describing the research object might spark misleading expectations about how the agent looks, speaks, or functions. Researchers further must decide whether the agent under study gets a name and, if so, whether the name is more human-like (e.g., “Alexa”; Etzrodt and Engesser 2021, p. 65) or more machine-like (e.g., “Voice Assistant”; Tassiello et al. 2021, p. 1072), and assignable to a gender (cf. Feine et al. 2020). Voorveld and Araujo (2020), for instance, have shown that interacting with a virtual assistant with a human (vs. no) name decreases autonomy concerns and increases persuasiveness.

The preceding overview has demonstrated that the specifics of communicative agents need to be considered at various stages of the study design. Against this background, we recommend that researchers carefully consider how they introduce the communicative agent to participants. Human-like names may be more appropriate for studying anthropomorphism, while machine-like names may be better if the research focus is not on the communicative agent. As it remains unclear if adopting or combining scales developed for humans or computers truly capture interactions with communicative agents, we need validated HMC instruments that consider the unique characteristics of communicative agents to be able to adequately compare results across different studies. Additionally, researchers should aim at conducting well-powered studies while controlling for participants’ different experiences with, knowledge of, and attitudes towards the communicative agent studied.

## Experiments in HMC research

Experimental methods are a central pillar of HMC research. In their systematic review across 18 communication-related journals, Liu et al. (2022) found that in the last decade, 61% of studies related to communication between users and technologies were experiments (p. 20). Experimental designs, conducted online or in the lab, generally come with certain benefits and challenges that need to be discussed before looking at more specific HMC-related challenges.

Experimental designs enable the testing of causal effects (cf. Chugunova and Sele 2020) and center on observing human behavior in situations and environments explicitly created for the study’s purpose (cf. Webster and Sell 2014). This makes them particularly suitable for investigating authentic but controlled interactions of users with communicative agents. Lab experiments further facilitate the combination of various data collection techniques (e.g., thinking aloud, interviews, participant observations), as well as objective (e.g., task completion time, log files, head-, eye- and finger-tracking) and subjective (e.g., self-report) measures.

When conducting experiments in the lab or online, HMC scholars increasingly rely on extended reality (XR) technology, including augmented and virtual reality, to systematically study human-agent interactions (cf. Voit et al. 2019; Ratcliffe et al. 2021). Extended environments minimize distractions and help participants focus on interacting with the communicative agent. They also allow for highly detailed observations as well as systematic control and manipulation of the study environment (Arntz et al. 2021; Kuliga et al. 2015). In addition, XR allows researchers to investigate (future-oriented) scenarios that are difficult to realize in the laboratory due to logistical, time, or financial constraints. In terms of measurements, XR technology enables detailed, objective, and unobtrusive assessments of user activity, including head, eye, and finger tracking and log data. On the downside, however, study participants may have difficulty handling the device and navigating the extended environment. Novelty effects may also bias the results. In addition, extensive technical knowledge is required to control the increasingly complex applications and to program and design authentic scenarios, which ultimately makes a careful cost-benefit calculation advisable.

Isolating the theoretical principle under study is a unique strength of experiments. However, to make this possible, participants are asked to follow clear instructions when communicating with an agent, ranging from restricted conversation duration to predefined tasks or question wordings. Artificial lab conditions or pre-constructed online scenarios can differ substantially from real-life interactions and carry the risk of measuring lab- or material-specific behavior that cannot be generalized across individuals, settings, or treatments (cf. Webster and Sell 2014).

Researchers also need to rethink the question of the control group. Face-to-face communication is still widely considered the gold standard of communication, mirrored in research designs in which interaction with a communicative agent is compared to a control group of human-human interaction (cf., for chatbots Beattie et al. 2020; Luo et al. 2019). However, studies in the spirit of a Turing test are increasingly called into question (cf. Spence 2019), and scholars have turned to comparisons between different types of communicative agents, for example to study the effects of social cues (cf. Go and Sundar 2019). Communicative agents are also compared to other media such as websites (cf. Ischen et al. 2020a, b; Whang and Im 2021) or search engines (cf. Gaiser and Utz 2022). Depending on the research interest, it may also be useful to compare the effects of interactions with a physically present agent with those of participants who watched a recording of this interaction (cf. Li 2015). Overall, no recommendation can be made as to what the “ideal” control group should look like, as this design choice always depends on the questions and contexts guiding the research activity.

### Experiments with simulated interactions

#### Pre-recorded material and vignette studies

Experiments investigating HMC often follow a demonstrational approach: Participants are exposed to pre-recorded screenshots, audio files, or videos showing a dialogue with an existing or fictitious communicative agent (cf., e.g., for text-based chatbots Beattie et al. 2020). For example, Voorveld and Araujo (2020) asked participants to imagine they invited guests for dinner and asked their voice assistant about an ingredient. A picture of the voice assistant was illustrated in this scenario before the pre-recorded answer was shown. Similarly, Song and Kim (2020) used video clips showing the human-like robot Pepper as a fashion advisor and then had participants rate its perceived characteristics.

Demonstrational approaches with pre-recorded materials and vignettes are well suited for conducting studies in an online environment with larger samples. They allow using authentic stimuli while having the maximum flexibility in manipulating them, as the stimuli can also contain material generated by the researchers themselves. However, with pre-recorded materials participants are only passive viewers of the interaction with the communicative agent. Hence, while this observer role provides a “unique view that people do not have when they are one of the interactants in a situation” (Abendschein et al. 2021, p. 307), no actual interaction between the participants and the agent takes place. As a result, factors such as (perceived) interactivity, which plays an important theoretical role when studying communicative agents (cf. Sundar et al. 2016; Ischen et al. 2020b), cannot be considered. Moreover, when comparing human agents and chatbots in vignette experiments, it can be difficult for participants to recognize the human agent as human since the chat interfaces of both agents look very similar, and the agents often only differ in their introduction and picture (cf., for chatbots Klein and Utz 2022). Generally, demonstrational approaches can affect the acceptance of and attitudes towards the communicative agent, as shown by Xu et al. (2015), who compared recorded vs. live interactions for human-robot interactions. Stimulus videos created from a first-person perspective might help users empathize with the scenario, somewhat remedying this shortcoming (cf., for voice-based agents Whang and Im 2021).

In summary, we recommend that researchers use pre-recorded material or vignette designs for cases in which they want to experimentally manipulate very specific factors and conduct experiments with large samples in an affordable way. However, depending on the type of communicative agent to be studied, the use of pre-recorded material may be less appropriate. Also, pre-recorded interactions are easier to implement when studying agents with low degrees of embodiment, such as chatbots, and harder to implement the higher the embodiment level. The central downside of this design is that participants are passive observers; pre-recorded materials are thus less suitable when aspects of the interaction (rather than characteristics of the agent) are to be studied.

#### The “Wizard of Oz” technique

Besides the use of pre-recorded materials and vignettes, HMC scholars rely on the so-called “Wizard-of-Oz” (WoZ) technique (cf. Dahlbäck et al. 1993). Here, study participants are led to believe that they are interacting with an autonomous agent when, in fact, a human (usually the investigator) operates the agent by remotely controlling its movements, gestures, or speech (cf. Riek 2012, p. 119). Like experiments with pre-recorded materials, WoZ gives researchers full control over the stimuli presented to participants. However, since participants interact with the communicative agent, WoZ studies are more resource-intensive (cf. Araujo 2020, p. 38) and thus more suitable for a lab environment and smaller samples. An exception are studies with text-based agents such as chatbots, which can easily be implemented in online experiments (cf. Westerman et al. 2019). The WoZ technique further allows to evaluate user responses to existing systems and working prototypes and can thus be of particular interest for researchers who want to examine advanced functionalities that commercially available robots are not yet capable of (Rietz et al. 2021, p. 2). However, this approach can also fuel false expectations about the actual capabilities of AI-controlled machines (cf. Riek 2012, p. 120).

From a conceptual perspective, WoZ at least partially transforms human-machine communication into human-human communication mediated by a “mechanical puppet” (Baxter et al. 2016, p. 393). Therefore, to obtain valid results and reduce human bias, the wizard must strictly adhere to a predefined interaction script, resulting in less authentic scenarios. Moreover, simulating the interaction modalities of the respective agent can be particularly challenging for agents relying on voice, movements, or gestures (cf., for social robots Chapa Sirithunge et al. 2018; Thunberg et al. 2021). Porcheron et al. (2020) thus developed a tool for voice-based WoZ studies called “NottReal”, a “cross-platform Python-based desktop application for Wizard-controlled voice interface studies, where the intent detection and slot filling of typical natural language interfaces is completed by a human operator” (p. 1). Similarly, Rietz et al. (2021) presented “a general and easy-to-use tool for the Pepper robot” (p. 1). However, applying the WoZ technique in studies on interactions with robots (vs. other agents) still requires considerable technical and coordination skills from communication scholars. Especially for multimodal interactions, it is difficult to control robot behavior in detail and between different trial sessions since individual operators for motion and speech are necessary. For studies with robotic agents, Walters et al. (2005) therefore recommend combining direct WoZ controls with autonomous behaviors and functions.

Finally, when studying voice-based agents, it is necessary to decide which type of voice the agent should have. On the one hand, researchers can choose voices from existing agents such as Amazon Alexa, for example, by using Amazon Blueprint Skills, where individual responses can be easily programmed and recorded (cf. Weidmüller 2022). Alternatively, one may opt for lesser-known artificial voices such as Microsoft’s text-to-speech voice Zira (cf. Yuan et al. 2019) or demo versions of online text-to-speech interfaces that offer different voices, languages, and effects for limited text input (e.g., https://ttsdemo.com/, used by Rhee and Choi 2020). Finally, there is the option of recording the response in a human voice—either one’s own or that of an actor.

To sum up, the WoZ method is the best choice for researchers who want to study interaction but still want full control over it. However, researchers must be aware that the interactions might not be free of human bias and do not represent authentic HMC. Simulating autonomous systems poses ethical problems, as participants are deceived and cannot identify who or what they are interacting with. Additionally, researchers must have the necessary time and resources—especially when planning well-powered studies. This method is also suitable for conducting future-oriented experiments (e.g., studying communication styles current agents are not yet capable of). Due to the high standardization of the study design, WoZ experiments promote replication by other researchers.

### Experiments with non-simulated interactions

To address the limitation of low external validity inherent in experiments with pre-recorded materials or the WoZ technique, researchers design experiments that involve non-simulated interactions with communicative agents. In principle, these experiments can be conducted online and in the lab. However, an online setting is ideal for research on text-based agents such as chatbots since participants necessarily interact with the agent via a phone or computer anyway. For voice-based agents with a low degree of embodiment, e.g., voice assistants installed on a phone or computer, the lab environment is advantageous because it is easier to have people speak to an agent in a supervised environment. Technical requirements for interactions such as working microphones and speakers can be configured in advance, preventing unnecessary drop-outs due to technical issues. This becomes even more crucial as the level of embodiment increases: Voice-based agents with physical elements such as smart speakers would require a video call for participants to see and interact with the speaker. The same holds for highly embodied communicative agents such as social robots, with the added risk that participants may not be able to recognize and interpret the robot’s movements and gestures correctly. Here, lab experiments have distinct benefits as participants can (physically) interact with the agent and experience their presence.

In the following, we address specific challenges in experimental designs with actual agents resulting from the distinct characteristics of self-created (i.e., researcher-created) and commercially available agents.

#### Experiments with self-created agents

Regardless of the modality, creating a communicative agent from scratch requires considerable effort and expertise and can be a “complex, lengthy, and costly endeavor, involving a host of computational techniques […]” (Porcheron et al. 2020, p. 1), and for many scenarios, supporting tools are readily available. For interactions with text-based agents, such as chatbots, for example, there is a wide range of commercial tools whose basic versions can be freely used. Examples include Google’s Dialogflow, IBM Watson, and Microsoft’s Azure Bot Service (cf. Adam et al. 2021). There are also free, open-source frameworks for building chatbots, such as Botpress and RASA Stack. However, because these tools were not primarily developed for research purposes, they might lack basic functionalities needed to conduct experimental research, such as setting up different experimental conditions or being able to log the conversations for analysis. To circumvent these issues, Araujo (2020) developed a user-friendly chatbot framework for conducting experimental research, which has been used in several online studies (cf. Araujo 2018; Voorveld and Araujo 2020; Ischen et al. 2020a, b). Nevertheless, communication scholars still need some programming expertise to create and implement a chatbot solution.

Additional challenges arise for interactions with voice-based agents, as they require tools that support spoken language. In the study by Chérif and Lemoine (2019, p. 34 f.), for instance, participants interacted with a voice assistant developed by a company specialized in creating conversational interfaces. If researchers aim to investigate embodied voice-based agents integrated into smart speakers or robots, the physical appearance requires consideration, too. One advantage of using a neutral physical appearance that does not resemble any existing technology (cf. Rhee and Choi 2020) is that people are not biased regarding appearance and do not link it to companies like Amazon or Google. However, a neutral appearance could also lead to possible skepticism due to unfamiliarity. Depending on the research question, it may also matter whether the agent is more humanoid or machine-like. Also, researchers again need to decide what kind of voice they want for their self-created agent, like synthesized voices or voices recorded by a human. Choosing one or the other could lead to the voice being perceived as unnatural or too human-like (cf. Perez Garcia and Saffon Lopez 2019).

Taken together, creating an agent for an experimental study allows having an authentic human-machine interaction and still having some control over the material. However, researchers should choose this option carefully because it requires the highest effort and considerable programming skills (cf. Grudin and Jacques 2019, p. 6). In line with other researchers (cf. Liu et al. 2022), we recommend cooperating with interdisciplinary partners to make the process more feasible. Further, researchers should critically evaluate whether a study’s research question warrants the creation of an agent or whether other methods are a reasonable alternative to answer the research question.

#### Experiments with commercially available agents

Experiments involving non-simulated interactions can also be performed with commercially available agents such as Amazon’s Alexa. The biggest advantage here is that existing chatbots and voice assistants already have the necessary software installed, and their procurement is rather effortless. The situation is somewhat different for agents with a higher degree of embodiment, such as social robots. In most cases, commercially existing robots only come with a specific skillset or include demo programs. For robots such as Pepper or NAO, researchers can purchase the physical element of the robot equipped with the necessary hardware and programming instructions, which can then be adapted for specific study purposes (cf. Silva et al. 2019). Nevertheless, researchers should be familiar with programming languages such as C++, Python, or Java.

Regardless of the type of agent, commercially available technologies entail restrictions that might conflict with the research aim. Most importantly, participants might experience misunderstandings and conversational breakdowns, which can bias the experimental results. Although they have evolved greatly in recent years, agents such as Amazon’s Alexa still have limitations, resulting in incorrect or unintended answers or the inability to solve certain tasks (cf. McTear et al. 2016). Investigators have, thus, no control over the materials or answers presented. However, if the research addresses the current use of communicative agents, present-day agents—including their flaws—represent the reality of users and are thus important research objects. Using existing smart speakers outside the user’s domestic environment, and with a non-personalized device, however, can affect the interaction. There is evidence that customizing voice preferences (cf. Tolmeijer et al. 2021), privacy settings, and contents (cf. Cho et al. 2020) can significantly influence attitudes towards and engagement with voice-based assistants. Consequently, prior experience with the agent under study can considerably affect the results, making it an important moderator (cf. Croes and Antheunis 2021).

Concerning chatbots, there are many existing agents to choose from, ranging from rather general or social ones (e.g., Cleverbot, Replika, or Kiku) to rather specialized ones (e.g., in customer service). In contrast, the range of available voice-based agents and social robots is rather small. Almost all the commercially available voice assistants depend on big tech companies like Amazon or Google and their algorithms (Natale and Cooke 2021), which the researcher has no control over. In all cases, researchers must ensure that the agent is reset to the same settings after each measurement to avoid bias from previous interactions. For social robots, the few commercially available products on the market often have limited skills (e.g., small home robots like Vector or Emo, Kellermayer et al. 2020) or are rather expensive (e.g., robotic pets AIBO and PARO, Carros et al. 2020).

In conclusion, using commercially available agents for research purposes can have benefits: obtaining the agents can be fast, easy, cost-effective, and less prone to errors. However, their limitations can conflict with the research aim, as researchers have significantly less control over the material and the interaction. Additionally, the number of available options varies with levels of embodiment and modalities.

## Field studies in HMC research

Several HMC researchers argue that more attention needs to be paid to situated, everyday use practices with communicative agents (cf. Fortunati and Edwards 2021, p. 23; Suchman et al. 2019, p. 14). Field studies refer to direct or indirect observations conducted in the user’s familiar environment (cf. Butz and Krüger 2017, p. 154). Direct observations entail analyzing audio or video recordings of interactions, ethnographical, and design studies. Indirect observations, by contrast, refer to analyzing diary entries, log files, or digital trace data after the observation (cf. Butz and Krüger 2017, p. 125ff.). Diary or experience sampling studies further allow investigating people’s use and perception of communicative agents over longer periods of time (cf., for voice assistants Mavrina et al. 2022). Here, people use a given system in their natural environment and document their experiences, perceptions, and feelings at certain times (diary study, cf., e.g., for chatbots Muresan and Pohl 2019) or in certain situations (experience sampling study, cf., e.g., for voice assistants Geeng and Roesner 2019). The documentation of those real-world experiences often happens in a structured format, e.g., via an online questionnaire or an app.

Both direct and indirect observations of users’ interactions with communicative agents can be obtained and analyzed quantitatively, qualitatively, or by using a mixed-methods approach combining several research methods. Crolic et al. (2022), for example, analyzed real-world data from a telecommunications company using statistical analysis in order to investigate the impact of human-like chatbots on customer responses. Tsiourti et al. (2020) conducted an ethnographic household-based study on the adoption of the robot Anki Vector during the COVID-19 pandemic using qualitative and quantitative field techniques.

Qualitative research in human machine communication also includes the co-creation of technological systems with users (cf. Axelsson et al. 2021; Nielsen et al. 2021). Such a participatory design prioritizes aspects like users’ diversity, competence, and autonomy. As such, it might address challenges regarding the size and composition of the samples studied, as not least in Germany, both access to automated communications technology and the skills to interact with this technology in a meaningful way are unevenly distributed across the population (cf. DESTATIS 2021). From a methodological perspective, this could lead to a self-selection bias and, subsequently, a narrowing to specific population segments. However, when relying on participatory design principles, it needs to be considered that inexperienced users may have problems talking about specific features or functionalities of communicative agents, as they cannot imagine communication and interaction with a technology they have never seen or used.

Overall, field studies ensure high external validity, allowing researchers to apply the findings to real-world use (cf. Schmidt et al. 2021, p. 2; Butz and Krüger 2017, p. 154 f.). Despite this indisputable advantage, field studies have rarely been applied in HMC research so far: Liu et al. (2022, p. 48) only found seven field observation studies in their systematic review of HMC research. Rapp et al. (2021, p. 6) only found five field studies in their systematic review on human-like features in text-based chatbots. This low number might stem from several methodological and practical challenges addressed below.

### General challenges of field studies in HMC research

Letting people interact with communicative agents without restriction comes with a significant loss of control over participants’ behavior—even more so in longitudinal designs. Unforeseen disturbances and distractions can negatively influence users’ motivation and concentration, which can result in lower internal validity (cf. Schmidt et al. 2021, p. 2). Researchers can remind participants regularly by sending them notifications to mitigate motivational problems. Larger incentives, such as allowing participants to keep a device given to them during the study (cf., for social robots de Graaf et al. 2016), might also enhance compliance.

A major challenge in field studies is the large amount of time, materials, organization, and skills required. For example, as log data of user interactions with communicative agents are often unstructured (e.g., text, audio, or video records), cleaning and analyzing them requires a lot of effort and oftentimes special software. Field studies also place high demands on the technology being studied, i.e., prototypes that function reliably outside the laboratory must be available to enable realistic operation in the actual user environment (cf. Schmidt et al. 2021, p. 2). To make sure that participants can interact with communicative agents without the researcher’s guidance, support needs to be available (cf. Schmidt et al. 2021, p. 2). Long-term studies require the technology to be even more robust.

Using robust prototypes for academic research often requires collaboration with an organization that can provide access to a system or users and their data (cf., e.g., Luo et al. 2019; Crolic et al. 2022). Organizations willing to cooperate with research institutions sometimes can be hard to find, particularly if researchers do not have many industry contacts. In addition, when collaborating with an organization, the interests of the researcher and the organization can differ. For example, a field experiment to study conversational errors of a chatbot selling products, where participants are real customers of a company, might not be approved by management due to expected revenue losses. Also, companies may be reluctant to release real customer data, e.g., collected via a customer service chatbot, for log analyses due to data security concerns.

### Field studies in private settings

Several privacy issues arise when conducting field research in users’ homes. First, to investigate user behavior towards communicative agents, behavioral (trace) data, including conversation logs, which might contain sensitive information, are collected and analyzed (cf., e.g., Bentley et al. 2018; who analyzed the logs of 65,499 interactions from 88 diverse homes). If a household receives, for example, a smart speaker for research purposes, all household members must consent to have their interactions with the agent recorded and analyzed. As recordings have implications from a data protection (GDPR) and research ethics perspective, researchers may face significant reservations from participants—especially over a longer period (cf. Hector and Hrncal 2020, p. 4 f.). Second, there is also the ethical perspective of bringing company devices into people’s homes and indirectly endorsing a technology and a brand that collects participants’ interaction data and stores it on their servers for their own purposes. These ethical concerns are exacerbated because commercially available smart speakers such as the Amazon Echo have been proven to record private interactions not directed to them (cf. Ford and Palmer 2019, p. 78).

Alternatively, data donations of people already using specific communicative agents can be obtained. For example, Sciuto et al. (2018, p. 859) recruited people in online forums who would share the log history of their Amazon Alexa by installing a browser extension. Bentley et al. (2018, p. 4) asked participants to donate their interaction history with their Google Home device. However, even if participants agree to donate their data, ethical aspects must be considered. For instance, smart speaker owners can only agree to donate recorded data for utterances of themselves. Any input by other household members or guests should be deleted from the donated data. Participants should also be informed about the possibility that the voice assistant recorded private conversations and given the opportunity to edit or delete data they feel uncomfortable sharing before donating (e.g., Bentley et al. 2018, p. 4). Regardless of the communicative agent, for ethical reasons, when interaction data are donated, researchers should indicate in advance that logs may contain highly sensitive or personal data (e.g., on health, sexual orientation, religion) so that participants are made aware of the risk. Careful and confidential handling of data, ideally with pseudonymization if not anonymization, should be a matter of course for researchers.

However, both approaches, equipping households with communicative agents and accepting data donations, are likely to lead to selection effects that reduce the generalizability of the results. (Early) adopters of communicative agents are likely to have different traits than non-adopters, e.g., they possess a higher acceptance of technological innovations and are technologically savvier (cf., for social robots Bernotat and Eyssel 2018). People who donate their interactional data thus differ from people who do not own a communicative agent yet. In addition, potential intervening variables like variations between devices or household scenarios should be considered when analyzing interaction records, as these might affect the communication with the agents and thus the study outcome (cf. Beneteau et al. 2020; Garg and Sengupta 2020).

### Field studies in public settings

In addition to the home environment, researchers have explored unconstrained interactions with communicative agents in public places such as a university (cf., for voice assistants Lopatovska and Oropeza 2018) or an exhibition (cf., for voice assistants Siegert 2020; for social robots van Straten et al. 2022). However, only a few or short interactions may be recorded here because the visitors are too shy to talk to the agent for longer or the agent does not understand their requests (cf. Lopatovska and Oropeza 2018, p. 315). Gamification elements might remedy this challenge (cf. Siegert 2020, p. 617). Regarding research on interactions of humans with embodied agents in the public sphere, video recordings of those interactions are often necessary to study, for example, the way people react to a robot in a train station (cf. Hayashi et al. 2007). Researchers must request permission from the authorities and ensure users know their interactions are recorded, e.g., via a notice on site.

In summary, field studies can provide unique insights into users’ experiences, perceptions, and evaluations of communicative agents in private or public settings and over time. Although they involve a great deal of organizational, financial, and data protection effort, researchers should aim to conduct more of them because “if you want to understand the big issues, you need to understand the everyday practices that constitute them” (Suchman et al. 2019, p. 14).

## Discussion and conclusion

In this paper, we have provided an overall overview of challenges typically encountered by communication scholars when designing and conducting empirical studies on people’s interactions with conversational agents. Specifically, we have synthetized and critically reflected on the benefits and downsides associated with dominant approaches in HMC research, namely experiments and field studies involving chatbots, voice assistants, or social robots. Yet, to move towards building theoretical frameworks central to HMC, diverse methodological and epistemological perspectives are necessary. By focusing on experiments and field studies we miss insights that could be gained when applying qualitative or mixed-method approaches. We therefore recommend for future research that similar overviews be developed for other empirical approaches, aiming to provide support and inspiration for those new to the field.

Methodological challenges need to be considered in the context of the research question to be answered. Our overview relates to the empirical study of interactions between humans and machines—an area of research that is vital for advancing (theoretical) debates on how humans consider communicative agents as social others. Experiments in particular are powerful tools for examining such processes in light of affordances provided and enacted through communicative agents. As displayed in Table 1, the main challenges of experimental research approaches are associated with issues of stimulus manipulation and validity. While this affects all communication research, it plays an even more significant role in HMC because creating high-quality stimuli in an authentic setting requires a skillset that goes beyond the traditional toolkit of communication scholars. This is especially true for studies involving agents with advanced levels of embodiment or communication abilities, calling for collaborations with computer scientists and media engineers. Hence, also from methodological perspectives, “the need for interdisciplinary dialogue among scholars is key to HMC” (Liu et al. 2022, p. 6). Although interdisciplinary collaboration often comes with obstacles, such as dealing with divergent ways of working and thinking, the benefits of combining skills from social science, humanities, and engineering can be well worth the extra effort.

As our paper has shown, there are nevertheless also a growing number of tools that communication scholars can use to study interactions between humans and communicative agents with relatively simple means. One such approach is the use of commercially available agents such as Amazon’s Alexa. While these are a convenient way to study the situational use of conversational agents in people’s everyday lives their use must be carefully considered, especially in field studies, and not least from a GDPR/ethics perspective. By collecting real-life data from authentic interactions in natural environments, field studies present great opportunities to investigate situated, everyday use practices with communicative agents. However, the large amount of time and resources like staff and technology still restrict researchers who want to conduct HMC field studies.

Furthermore, HMC research differs from more classical research in communication not only because of the technical characteristics of the objects under study. Communicative agents are designed (and frequently perceived) as communication partners, anthropomorphized, and attributed with social presence. For many people, the technologies are at the same time very new, even futuristic. Therefore, more so than with other technologies, empirical designs need to pay attention to intervening factors linked to perceptions and attitudes toward artificial intelligence/robotics or to inherent social cues such as the voice or name of a voice assistant.

Taken together, we recommend that HMC researchers collaborate with researchers from other disciplines, be open to trying new methodological approaches, consider differences in people’s knowledge, experiences, and attitudes towards communicative agents, be highly transparent when discussing the method in a publication, and share their experiences with other researchers in the field. The attention paid to new social technologies such as chatbots, voice assistants, or social robots from a communication science perspective has increased greatly in recent years (cf. Richards et al. 2022), and early-career researchers have begun being formally trained in HMC. Hence, besides learning the theoretical specifications of communicative agents, they also need the appropriate methodological tools to conduct good research. This paper would, therefore, also like to offer a first impulse to think about methods education in the light of the rapid technological development and our task to study them in a future-oriented way—in Germany and beyond. Overall, our paper does not claim to be exhaustive but rather aims to provide guidance and to serve as a starting point for discussions in communication science on how interaction between humans and machines can be investigated in the most adequate ways.
